# Phylogenetic History and Adaptation Mechanisms of *Casuarina equisetifolia* Landraces in China

**DOI:** 10.1002/ece3.72781

**Published:** 2026-01-05

**Authors:** Jingxiang Meng, Yong Zhang, Yongcheng Wei, Wei Lin

**Affiliations:** ^1^ Research Institute of Tropical Forestry (RITF) Chinese Academy of Forestry Guangzhou People's Republic of China

**Keywords:** adaptive mechanisms, *Casuarina equisetifolia*, genome, hybridization, phylogenetics

## Abstract

Following its introduction to China, *Casuarina equisetifolia* rapidly adapted to its new habitat and was extensively cultivated as a crucial tree species for coastal shelterbelts. However, the systematic classification of Chinese landraces remains controversial and the mechanism underlying the rapid adaptation of this species in China remains unknown. To understand more fully the phylogenetic history and adaptation mechanisms of *C. equisetifolia* , 66 samples were collected from Chinese landraces and five from other potentially related provenances. Single nucleotide polymorphisms were detected through genotyping by sequencing and used for genetic structural analysis and genome comparison. The genetic structures of Chinese landraces were largely consistent with that of *C. equisetifolia* in natural and other distributions, while 76.7% of individuals showed clear admixture with *Casuarina glauca* , indicating frequent spontaneous interspecific introgression or hybridization. Phylogenetic and gene flow analyses further support a distinct local origin involving unique parental combinations. Genomic variation associated with pathogen–plant interactions was observed between natural provenances and founder populations along migration pathways. Moreover, integrated genomic signals were identified in hybrids, including secondary metabolite synthesis derived from *C. glauca* , as well as growth regulation and metabolic processes inherited from *C. equisetifolia* , offering preliminary insights into adaptive mechanisms during the introduction process. These findings enhance our understanding of the phylogenetic history of *Casuarina* landraces in China and may contribute to improved efficiency in domestication and breeding programs.

## Graphical abstract

Genetic analysis revealed that Chinese *Casuarina equisetifolia* landraces exhibit structures similar to Southeast Asian populations but also show evidence of hybridization with *C. glauca* . The study detected genomic variations linked to pathogen interactions along migration pathways and identified integrated adaptive signals in hybrids, including traits from both parent species related to secondary metabolites and growth regulation. These findings clarify the phylogenetic history of Chinese casuarina landraces and provide insights into their rapid adaptation, potentially aiding future domestication and breeding efforts.

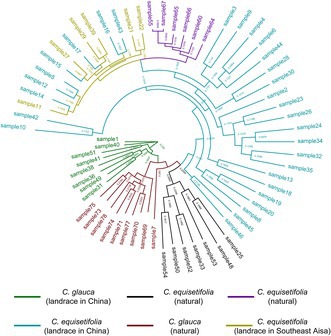

## 1 Introduction

The artificial introduction of species to new habitats allows humans to utilize plant resources but also facilitates their dissemination and evolution (Schreiber et al. 2024; Escandon 2022). Unlike natural evolution, that following artificial introduction often involves more complex selection pressures imposed by both the new habitat and human requirements, thereby increasing the risk of failure or loss of control (Gioria et al. 2023; Maurel et al. 2016). Successful instances of artificial introduction and utilization tend to attract attention from breeders because understanding the mechanisms underlying rapid adaptation in such cases may guide future work and enhance the efficiency of domestication and artificial breeding programs (Schreiber et al. 2024; Maunder 1992).

Casuarinaceae is a family of woody species comprising four genera and more than 90 species (Steane et al. 2003). Most of these species expanded naturally across Oceania, providing vital plant resources for landscaping, fuelwood, pulp production, and ecological forestry (Potgieter et al. 2014; Vikashini et al. 2018). Over the past two centuries, *Casuarina* has been widely introduced to tropical and subtropical regions, forming many landraces with significant variation from the natural provenances (Zhang et al. 2020; Van der Moezel et al. 1989). China has the northernmost distribution area of *Casuarina* along the Pacific Rim. Since its initial introduction in the 1890s, large‐scale domestication and cultivation of *Casuarina* have occurred in three main periods: around 1900, during 1950–1960, and after 1980 (Zhong et al. 2005). To date, over 20 *Casuarina* species have been evaluated in field trials, but only two species are now widely cultivated, with *Casuarina equisetifolia* forming forests at low latitudes in subtropical regions and *Casuarina glauca* distributed at slightly higher latitudes (Zhong et al. 2010).

In southern China, landraces commonly regarded as *C. equisetifolia* are of particular concern due to their extensive use in coastal shelterbelt construction, and there is currently no suitable substitute species available (Zhong et al. 2010; Tiwari and Talreja 2023). In most field trials, germplasms from these landraces are prioritized as superior materials for afforestation and breeding programs due to their enhanced growth rates, cold resistance, and disease resistance compared to germplasms transplanted from other regions (Ye et al. 2019; Xu et al. 2022). However, despite the widespread utilization of these germplasms in plantations and their importance as breeding resources, the systematic classification of Chinese *C. equisetifolia* landraces remains controversial, and the mechanisms by which they have adapted to this habitat remain poorly understood. As a crucial breeding resource utilized in global breeding programs (Pinyopusarerk et al. 2005), study of phylogenetic history and adaptation mechanisms is essential and will undoubtedly hold significant value for future research and applications.

Early studies based on simple sequence repeat markers (SSR) revealed a close genetic relationship between *C. equisetifolia* populations in China and Southeast Asia, indicating that species expansion had occurred gradually across geographic regions (Zhang et al. 2020; Hu et al. 2016). However, these findings were based on limited samples with typical species characteristics, constraining their explanatory power for entire populations. In reality, landraces classified as *C. equisetifolia* in China exhibit substantial genetic diversity, with some individuals even displaying traits similar to those of other species (Zhong et al. 2005). Previous studies have proposed interspecific introgression or hybridization as mechanisms for generating diversity (Varghese et al. 2004; Ho and Lee 2011; Pauldasan et al. 2023), but direct DNA evidence confirming widespread introgression in this region is lacking. Moreover, hybridization events and founder effects can significantly influence the adaptability of offspring (Dagilis et al. 2019). Previous studies have examined adaptive divergence among geographic populations (Hu et al. 2016), but have rarely explored the genetic foundations underlying adaptive variation. For these extant landraces, the mechanisms underlying rapid adaptation may be complex and difficult to fully elucidate using only simple molecular markers.

To understand the phylogenetic history and adaptation mechanisms of the controversial landraces more clearly, we selected 30 individuals commonly regarded as *C. equisetifolia* in China, along with five reference landraces from different provenances that may be associated with Chinese landraces. Whole genome analysis was conducted using the genotyping by sequencing (GBS) approach (Ning et al. 2025) to obtain a comprehensive result based on a large number of single nucleotide polymorphisms (SNPs). The main objectives of this study were (1) to evaluate spontaneous introgression or hybridization in *C. equisetifolia* landraces in China and reconstruct their phylogenetic history via structural analysis, and (2) to investigate the roles of founder effects and hybridization on *C. equisetifolia* adaptation along migration pathways based on genomic comparisons among related populations. Our findings offer valuable insights into the adaptation mechanisms of existing landraces in China, and may enhance the efficiency of domestication and breeding programs.

## 2 Materials and Methods

### 2.1 Study System

The sampling sites along coastal regions in China were randomly selected to account for the fragmented distribution of *Casuarina* landraces. To minimize the inclusion of clonal individuals, which have been predominantly planted over the past 20 years, only trees older than 20 years and located more than 100 m apart were included in the genomic analysis. The tested samples comprised individuals exhibiting diverse traits but that were previously broadly categorized as *C. equisetifolia* . Photosynthetic branchlets, which function as leaves in this species, were collected directly from the selected trees and stored at −20°C prior to DNA extraction.

**FIGURE 1:**
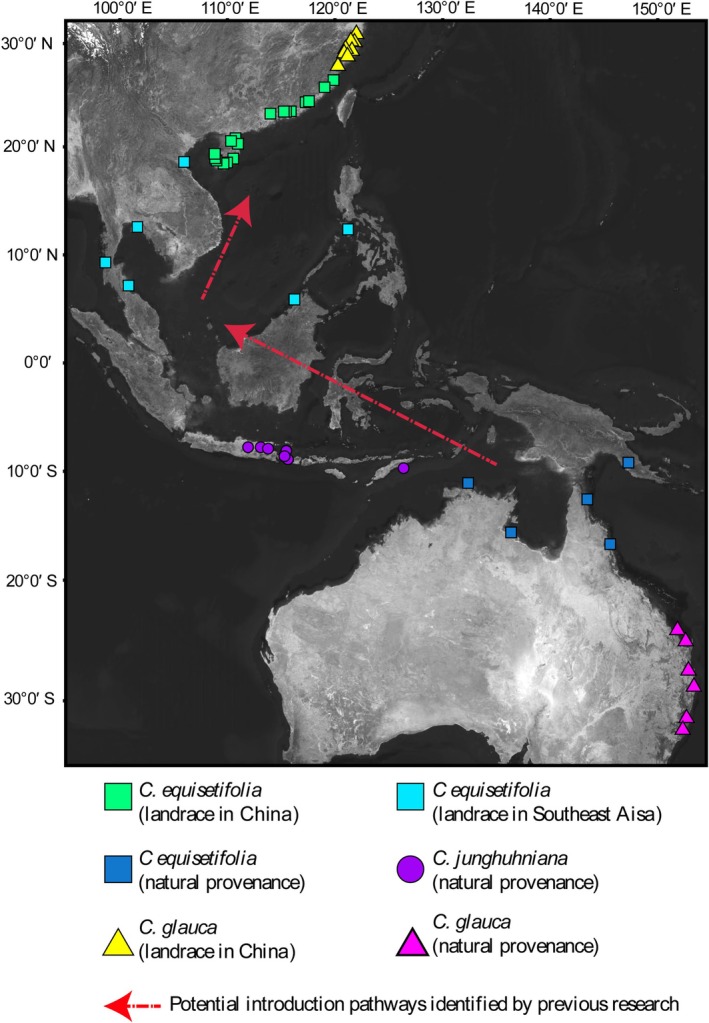
Collection localities of samples or seeds and the introduction pathways inferred from previous research (Zhang et al. 2020; Luo et al. 2007)

Reference plants were cultivated from seeds provided by the Commonwealth Scientific and Industrial Research Organisation in Australia. These seeds included three species of natural provenance ( *C. equisetifolia* , *C. glauca* , and *Casuarina junghuhniana*) and two Southeast Asian landraces ( *C. equisetifolia* in Southeast Asia and *C. glauca* in China). These species are distributed along proposed *C. equisetifolia* introduction pathways (Zhang et al. 2020; Luo et al. 2007), and their fertility rates have been determined through artificial hybridization experiments (Zhang et al. 2016). After removing duplicate samples collected from the same stands, a total of 66 individuals were obtained for genomic analysis. Geographic information for all samples is provided in Figure 1 and Table S1.

### 2.2 DNA Extraction

A modified cetyltrimethylammonium bromide method was employed to extract DNA from photosynthetic branchlets, as previously described (Meng et al. 2023). DNA from each of the 66 samples was resuspended in 100 μL of tris‐ethylenediaminetetraacetic acid buffer solution. After concentration and quality testing, 200 ng of DNA was carefully extracted from each sample for library construction.

### 2.3 Library Construction and Genome Sequencing

Genomic DNA was incubated at 37°C with MseI endonuclease, T4 DNA ligase, and adenosine triphosphate (New England Biolabs, Ipswich, MA, USA), and an MseI Y adapter containing a barcode. The restriction ligation reactions were inactivated by heating at 65°C, followed by digestion at 37°C using MseI and NlaIII restriction enzymes. The samples were purified using Agencourt AMPure XP beads (Beckman Coulter, Brea, CA, USA) and subjected to polymerase chain reaction (PCR). Fragments of 375–400 bp were isolated using a gel extraction kit (Qiagen, Hilden, Germany), repurified, and diluted for sequencing.

The library was sequenced using Illumina HiSeq PE150 and clean data obtained by trimming adapter sequences; low quality reads were aligned against the reported *C. equisetifolia* genome (Zhang et al. 2023) using the Burrows Wheeler Aligner, with the parameters “mem ‐‐ t4 ‐‐k32 ‐‐M”. SNPs were identified using SAMtools (Li et al. 2009; Li 2011). Quality filtering was conducted using VCFtools (Danecek et al. 2011), retaining SNPs with a missing rate < 10%, minor allele frequency > 0.05, and only biallelic loci.

### 2.4 Genetic Structural Analysis Based on Landrace Provenance

To understand the background of the landrace provenances, structural analysis was performed using structure software (Pang and Zhang 2025). The backgrounds of the landrace samples were estimated through comparison with the five reference provenances.

Phylogenetic relationships among the tested samples were analyzed using the SVDQuartets analysis (Vachaspati and Warnow 2018) implemented in PAUP*(Version 4.0a169), and the gene tree was visualized based on the SVDQuartets results using the ggplot2 (Gómez‐Rubio 2017) package in R software (Version R‐4.1.1).

To investigate gene flow among the tested provenances, ABBA–BABA test (Kong and Kubatko 2021; Frankel and Ané 2023) was employed by assessing *D* statistics, with *C. junghuhniana* designated as the outgroup. In each analysis, when a landrace of *C. equisetifolia* or *C. glauca* was set as P2, the corresponding natural provenance was used as P1.

### 2.5 Diversity Analysis Among and Within Cluster Populations

Based on the structural analysis results, the tested individuals were reclassified to divide *C. equisetifolia* samples from China into different taxa. Principal component analysis (PCA) was also conducted using R‐4.1.1 software; and the hierfstat package (Goudet et al. 2015) was used to verify the reliability of the results. Samples with different genetic backgrounds were further subgrouped to understand more clearly variation within each provenance.

Analysis of molecular variance was conducted to evaluate the hierarchical partitioning of genetic variance among cluster groups using the poppr package (Kamvar et al. 2014) in R based on 10,000 permutations. Genetic diversity parameters for each cluster or provenance were calculated using VCFtools (Danecek et al. 2011), including nucleotide diversity (Pi), observed heterozygosity (Ho), unbiased expected heterozygosity (He), the fixation index (Fis = 1 − Ho/He), and Tajima's *D*. To evaluate genetic correlations among the tested provenances, the pairwise fixation index (FST) was calculated between each pair of groups using the adegenet package (Kamvar et al. 2014) in R‐4.1.1.

### 2.6 Genomic Comparisons Among Related Populations

To investigate the impact of selection on the genetic structure of different populations, we identified SNPs with significant changes in allele frequencies as signals of selection (FST > 0.6).

Genome fragments encompassing the selected SNPs (±1000 bp) were extracted from the *C. equisetifolia* genome. Gene prediction was performed by aligning these fragments to the *Arabidopsis thaliana* genome, as no annotation file has yet been created for the *C. equisetifolia* genome. The functions of genes within these fragments were further characterized using Gene Ontology (GO) (Consortium GO 2004) and Kyoto Encyclopedia of Genes and Genomes (KEGG) analyses (Kanehisa et al. 2025). All computational procedures were conducted on the Galaxy Online Platform (https://usegalaxy.cn/).

## 3 Results

### 3.1 Genetic Structure of the Tested Provenances

A total of 104,232,164 sequencing reads were generated from the 66 individuals, with an average sequencing depth (X) ranging from 11.99 to 14.02 across samples. Based on SNP calling in SAMtools, 796,396 SNPs were detected, of which 13,591 met the quality requirements and were used for further analysis.

**FIGURE 2:**
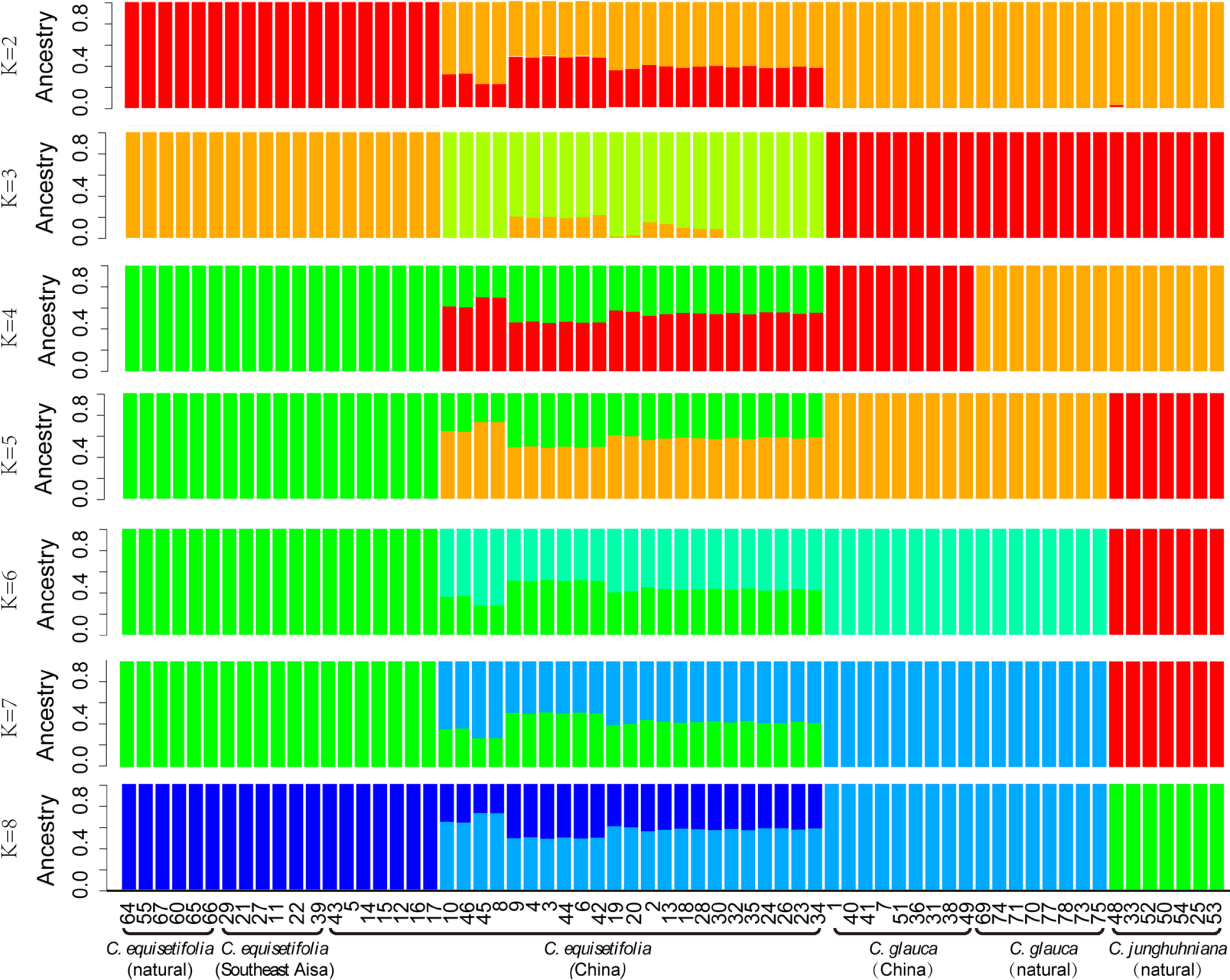
A structure plot of the test samples from *K* = 2 to *K* = 8.

Structure analysis for *K* values ranging from 2 to 8 is presented in Figure 2. When *K* ≥ 5, natural provenances of *C. junghuhniana* , *C. glauca* , and *C. equisetifolia* were successfully distinguished, and the *C. equisetifolia* in Southeast Asia and *C. glauca* in China cluster with their respective natural provenances. *C. equisetifolia* in China was divided into two subgroups: seven individuals were clustered with the *C. equisetifolia* group, while 23 others showed a mixed genetic structure intermediate between the *C. equisetifolia* and *C. glauca* groups.

**FIGURE 3:**
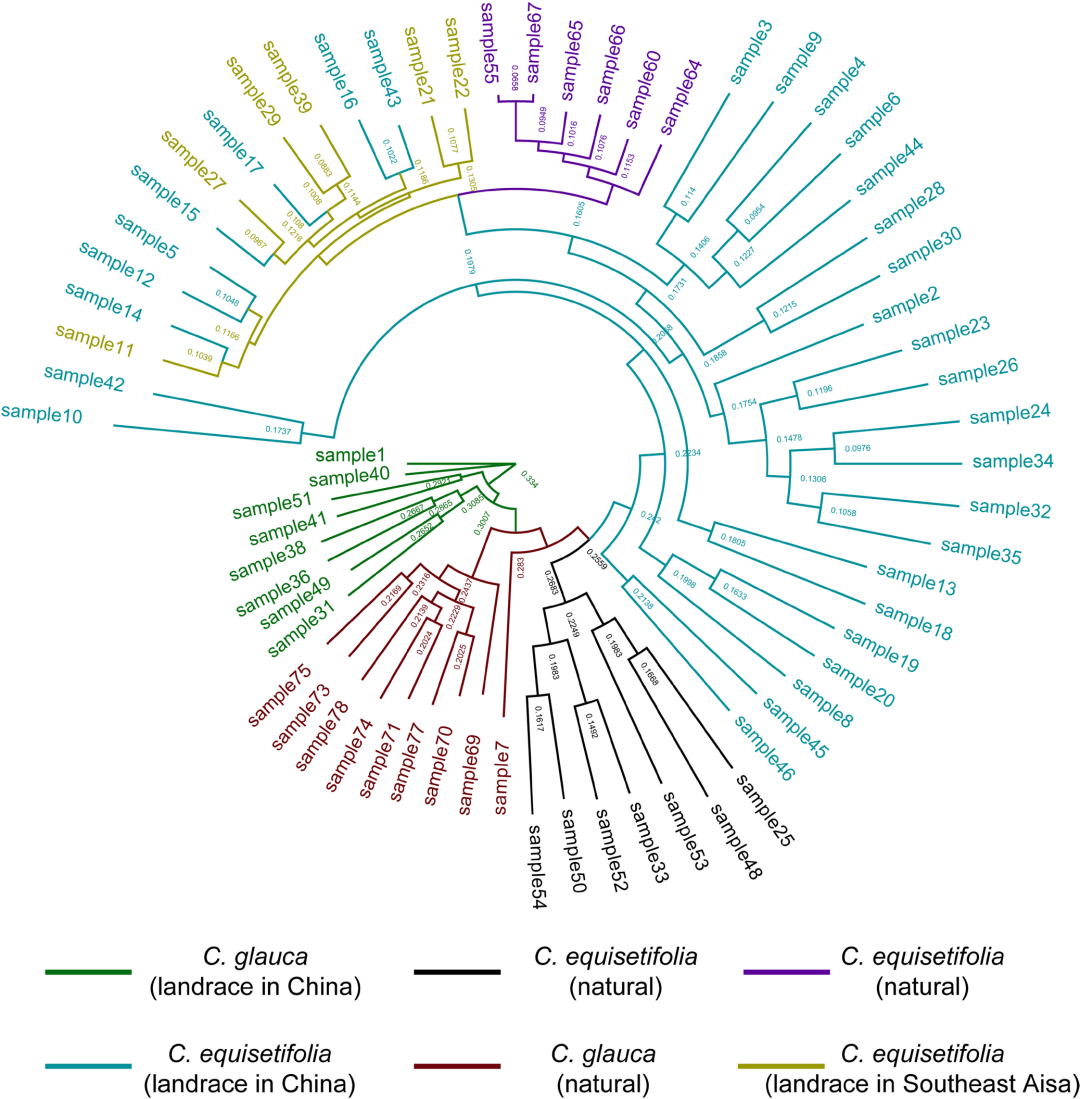
Phylogenetic analysis of the test samples.

Phylogenetic analysis of 66 individuals (Figure 3) reveals distinct clades corresponding to three species. *C. equisetifolia* in Southeast Asia is closely related to the respective natural provenance but constitutes an independent taxon. Seven individuals of *C. equisetifolia* in China cluster with the samples from Southeast Asia, indicating genetic affinities. In contrast, the remaining 23 individuals form a distinct clade that is positioned intermediately among the three species and exhibits discernible internal substructure. Phylogenetically, *C. equisetifolia* is more closely related to *C. junghuhniana* than to *C. glauca* and may be genetically more divergent from the landrace of *C. glauca* in China compared to the natural provenances.

Analysis of *D* statistics reveals significant gene flow (*p* < 0.001) between provenances of *C. equisetifolia* in China and in Southeast Asia, as well as between *C. equisetifolia* and *C. glauca* within China, suggesting potential hybridization or introgression among these taxa rather than direct ancestry from natural provenances. In contrast, the gene flow between *C. equisetifolia* in Southeast Asia and *C. glauca* in China is not statistically significant, indicating limited genetic exchanges between these provenances (Table 1).

**TABLE 1:** Analysis of *D* statistics among the tested provenances.

Index	P1	P2	P3	Dstatistic	*p*
1	*C. equisetifolia* (Natural provenance)	*C. equisetifolia* (China)	*C. glauca* (China)	0.349	< 0.001
2	*C. equisetifolia* (Natural provenance)	*C. equisetifolia* (Southeast Asia)	*C. equisetifolia* (China)	0.364	< 0.001
3	*C. equisetifolia* (Natural provenance)	*C. equisetifolia* (Southeast Asia)	*C. glauca* (China)	0.013	0.59
4	*C. glauca* (Natural provenance)	*C. glauca* (China)	*C. equisetifolia* (China)	0.048	< 0.001

### 3.2 Genetic Diversity Among and Within Provenances

According to the result of structural analysis and geographical distribution, the tested samples were classified into six taxa (Table 2): 13 samples of *C. equisetifolia* in Southeast Asia and China (CESC), 9 samples of *C. glauca* in China (CGC), 8 samples from the natural provenance of *C. glauca* (CGN), 6 samples from the natural provenance of *C. equisetifolia* , 7 samples from the natural provenance of *C. junghuhniana* (CJN), and 23 hybrid samples in China (HY).

**TABLE 2:** Reclassified populations for genetic diversity analysis.

Claster	Population	Tested samples								
1	Natural provenance of *C. junghuhniana* (CJN)	25	33	48	50	52	53	54		
2	Landrace of *C. glauca in China* (CGC)	1	7	31	36	38	40	41	49	51
3	Natural provenance of *C. glauca* (CGN)	69	70	71	73	74	75	77	78	
4	Landrace of *C. equisetifolia* in Southeast Asia and China	5	11	12	14	15	16	17	21	22
	(CESC)	27	29	39	43					
5	Natural provenance of *C. equisetifolia* (CEN)	55	60	64	65	66	67			
6	Hybrids in China	2	3	4	6	8	9	10	13	18
	(HY)	19	20	23	24	26	28	30	32	34
		35	42	44	45	46				

**FIGURE 4:**
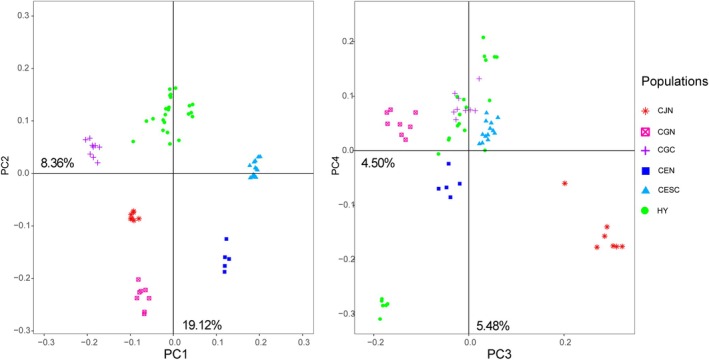
PCA analysis of the first four components of the test 6 provenance. CJN represent natural provenance of *C. junghuhniana* , CGN represent natural provenance of *C. glauca* , CGC represent landrace of *C. glauca* in China, CEN represent natural provenance of *C. equisetifolia* , CESC represent natural landrace of *C. equisetifolia* in Southeast Asia, HY represent hybrid found in China.

Significant genetic differences among the classified populations (*p* < 0.05) were observed in the AMOVA analysis (Table 3), and these classification results were further validated by principal component analysis (PCA), in which the first four principal components (PCs) accounted for 37.46% of the total variance (Figure 4). Based on the first two PCs, the samples were distributed across six distinct clusters: CGN, CEN, and CJN formed separate clusters in the lower portion of the graph; CGC was located in the upper‐left region; CESC occupied the right side; CESC individuals clustered in the left‐central area; and HY samples were positioned near the upper center. When the third and fourth PCs were considered, the HY population exhibited further differentiation, with five individuals located in the lower‐left quadrant and the remaining sample situated in the middle‐upper region.

**TABLE 3:** Analysis of molecular variance (AMOVA) of the 6 classified populations.

	df	Sum Sq	Mean Sq	Sigma	*p*
Among classified populations	5	6449.061	1289.8123	113.5968	0.0001
Within classified populations	60	6586.952	109.7825	109.7825	
Total	65	13036.014	200.5541	223.3793	

*Note:* degrees of freedom (df), sum of squares (Sum Sq), mean square (Mean Sq), and variance components (Sigma), which quantify the absolute genetic variation at each hierarchical level. The significance of these components was assessed using *p*‐values; a value below 0.05 indicates significant genetic differentiation at that hierarchical level.

The genetic diversity results for the 6 classified taxa are provided in Table 4. The *π* values for each population ranged from 0.045 to 0.184, with the highest values observed in CJN and the lowest in CEN. Ho values varied from 0.019 to 0.245, whereas He values ranged from 0.057 to 0.182. *F* values were negative in CGC, CESC, and HY and positive in the other taxa. Tajima's *D* values were positive for all 6 taxa, suggesting selection effects in the tested materials.

**TABLE 4:** Genetic diversity indices of the 6 classified populations.

	Ho	He	Fis	Tajima *D*	Pi
CJN	0.169	0.186	0.09	0.675	0.184
CGC	0.182	0.152	−0.2	0.409	0.153
CGN	0.056	0.123	0.547	0.526	0.114
CESC	0.088	0.078	−0.21	0.612	0.078
CEN	0.019	0.057	0.668	0.88	0.045
HY	0.245	0.182	−0.342	0.883	0.184
Total	0.121	0.129	0.07	0.303	0.192

*Note:* Pi denotes nucleotide diversity, Ho represents observed heterozygosity, He refers to unbiased expected heterozygosity, Fis is the fixation index calculated as Fis = 1 − Ho/He, and Tajima's *D* is a statistical test for deviations from neutral evolution.

The lowest FST value (0.034) was between CESC and CEN, while the highest value (0.166) was between CEN and CGC (Table 5). The pairwise FST value between CGC and CESC (0.156) was higher than that between CGN and CEN (0.153), suggesting further divergence between these two provenances.

**TABLE 5:** Pairwise fixation indices (FST) among the tested 6 provenances.

	CJN	CGC	CGN	CESC	CEN	HY
CJN	/	0.079	0.103	0.141	0.152	0.078
CGC	0.079	/	0.06	0.156	0.166	0.051
CGN	0.103	0.06	/	0.153	0.153	0.081
CESC	0.141	0.156	0.153	/	0.034	0.048
CEN	0.152	0.166	0.153	0.034	/	0.068
HY	0.078	0.051	0.081	0.048	0.068	/

*Note:* The FST value ranges from 0 to 1, with higher values indicating a greater degree of genetic differentiation.

### 3.3 Genetic Variation Along Introduction Pathways

**FIGURE 5:**
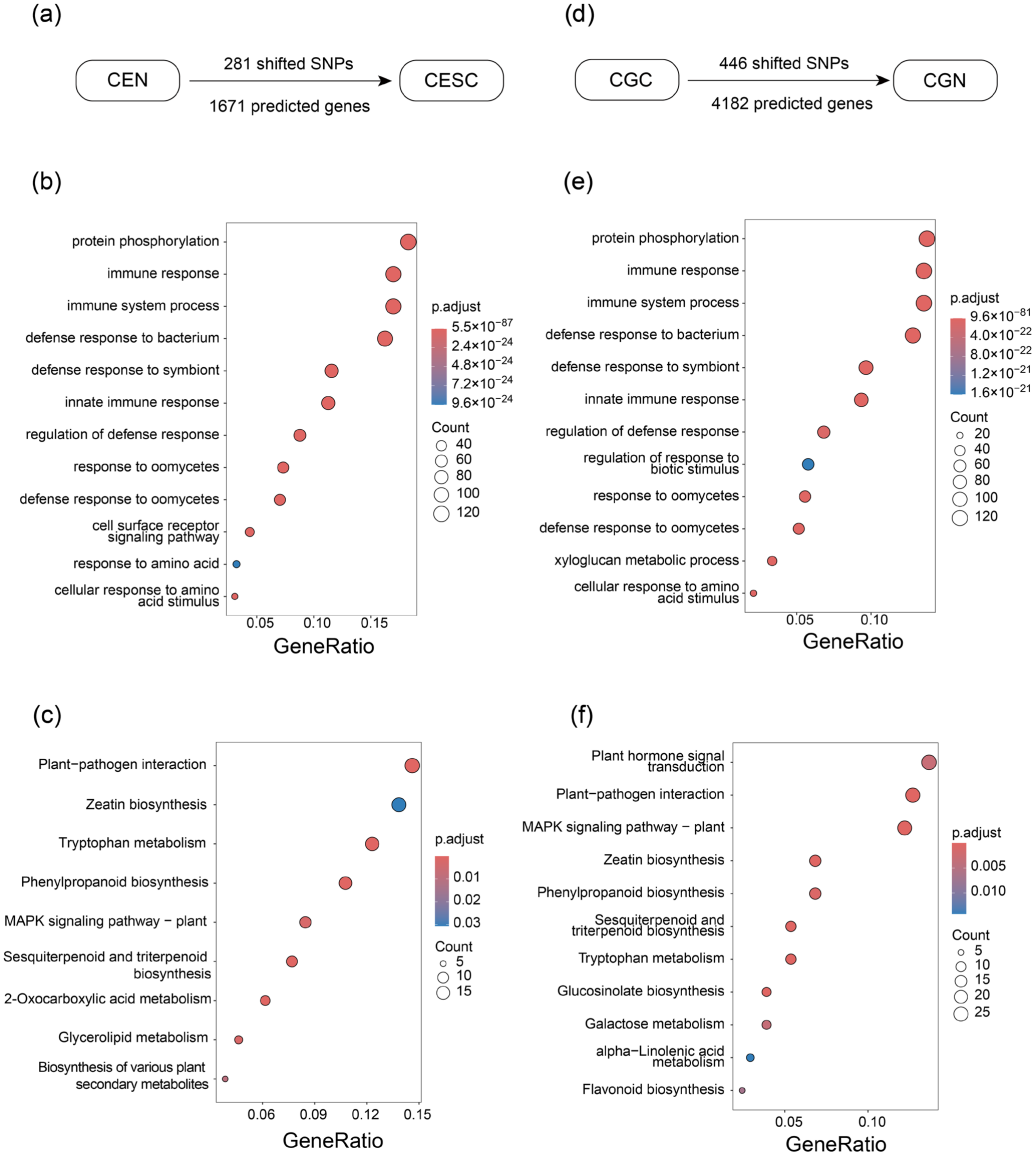
Genetic variation along species introduction event of *C. equisetifolia and C. glauca*. (a) Number of divergent SNPs (FST > 0.6) and predicted genes between CEN and CESC; (b) GO analysis of divergent SNPs from CGC vs. CGN; (c) KEGG analysis of divergent SNPs from CGC vs. CGN; (d) Number of divergent SNPs (FST > 0.6) and predicted genes between CGN and CGC; (e) GO analysis of divergent SNPs identified in CGC vs. CGN; (f) KEGG analysis of divergent SNPs identified in CGC vs. CGN.

SNPs with clear frequency shifts in paired populations (FST > 0.6) are presented in Figure 4. A total of 281 shifted SNPs were identified between CESC and CEN, with 1671 predicted genes located within the ±1000 bp region flanking these loci (Figure 5a). GO analysis revealed that these genes were predominantly associated with protein phosphorylation, immune responses and their regulation, immune system processes, defense response to bacteria, symbiont interaction, innate immunity, oomycete defense mechanisms, amino acid signaling pathways, and cellular responses to amino acids (Figure 5b). KEGG analysis further demonstrated that these genes were primarily involved in plant–pathogen interactions, zeatin biosynthesis, tryptophan metabolism, phenylpropanoid biosynthesis, and sesquiterpenoid and triterpenoid biosynthesis (Figure 5c). Additionally, 446 shifted SNPs were detected between CGC and CGN (Figure 5d), with 4182 predicted genes that were mainly involved in protein phosphorylation, immune‐related processes, bacterial and symbiont defense, innate immunity, biotic stress regulation, oomycete resistance, xyloglaucan metabolism, and cellular responses to amino acids (Figure 5e). KEGG analysis indicated that these genes were significantly enriched in pathways related to plant hormone signal transduction, plant–pathogen interactions, MAPK signaling pathway and zeatin, phenylpropanoid, sesquiterpenoid and triterpenoid biosynthesis (Figure 5f).

**FIGURE 6:**
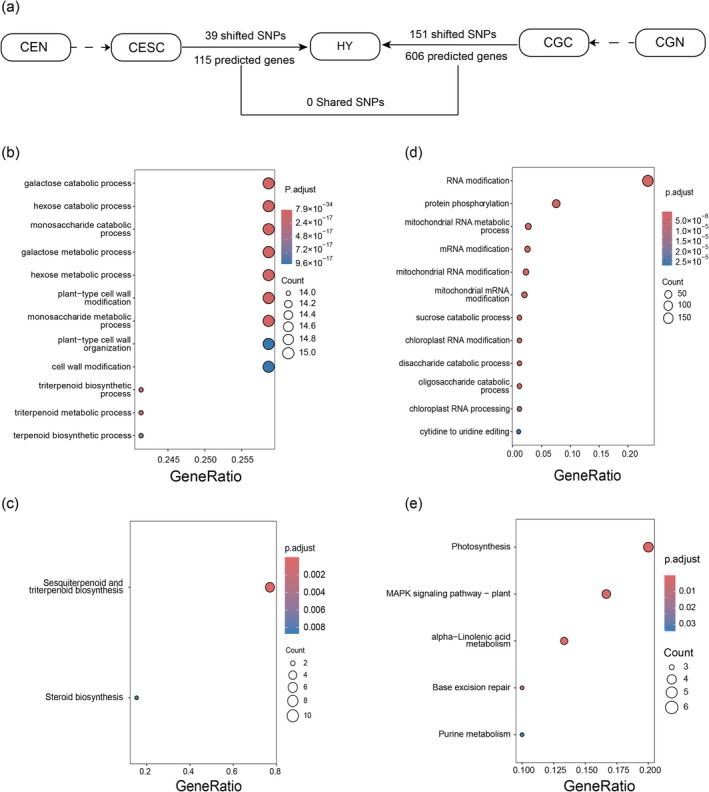
Selective signals during the hybridization process between *C. equisetifolia* and *C. glauca* . (a) Number of divergent SNPs (FST > 0.6) between HY and CESC and between HY and CGC; (b) Gene Ontology (GO) analysis of 39 SNPs identified in HY vs. CESC; (c) Kyoto Encyclopedia of Genes and Genomes (KEGG) analysis of 39 SNPs from HY vs. CESC; (d) GO analysis of 151 SNPs from HY vs. CGC; (e) KEGG analysis of 151 SNPs from HY vs. CGC.

Evaluating the hybrid populations in terms of their potential parental origin, only 39 distinct SNPs were identified between the hybrids and CESC, while 151 were detected between hybrids and CGC (Figure 6a). No shared selective signals were observed in these two comparisons, indicating that selection played a limited role apart from facilitating interspecific gene recombination. Based on GO analysis, the 115 predicted genes in HY vs. CESC were associated with functions such as galactose, hexose, and monosaccharide catabolic processes; galactose, hexose, and monosaccharide metabolic processes; and plant‐type cell wall modification and organization (Figure 6b). In contrast, differentially expressed genes between HY and CGC were predominantly related to processes including RNA modification, protein phosphorylation, mRNA modification and metabolism; sucrose, disaccharide, and oligosaccharide catabolic processes; and chloroplast RNA modification (Figure 6e). These results suggest that the hybrids may have inherited genes related to cell wall modification and monosaccharide metabolism from *C. glauca* , and those related to RNA modification, protein phosphorylation, and disaccharide catabolism from *C. equisetifolia* .

## 4 Discussion

### 4.1 Hybridization, Origins, and Adaptive Drivers

Landraces identified as *Casuarina equisetifolia* have long been recognized for their distinct phenotypic traits (Zhong et al. 2010). Previous studies have hypothesized the occurrence of natural interspecific hybridization in China; however, until now, this hypothesis has largely relied on indirect evidence derived from photosynthetic characteristics, seed morphology (Zhang 2013), and population level molecular markers such as RAPD and ISSR (Ho and Lee 2011). This study provides the first direct, individual level genomic evidence to resolve this longstanding debate, confirming natural hybridization between *C. equisetifolia* and *C. glauca (*Consortium GO 2004 *)* . Through comprehensive sampling and high resolution genomic analysis, we reveal a remarkably high prevalence of genetic admixture: 23 out of 30 individuals examined (76.7%) exhibit significant introgression from *C. glauca* , indicating that hybridization is not an isolated event but a widespread and recurrent evolutionary process in China. Notably, all admixed individuals sampled were mature trees exceeding 20 years of age, having endured prolonged exposure to environmental challenges, including typhoons, drought, pests, and diseases. The high proportion of hybrid individuals within this landrace suggests enhanced survival fitness, implying that interspecific hybridization may confer substantial adaptive advantages in the novel habitat. These findings are consistent with documented patterns of hybridization in *Casuarina* species across subtropical and southern subtropical regions, including reported populations in Florida, USA (Gaskin et al. 2009), Egypt (Badran et al. 1976) and some introduction area in Australia (Ghosh et al. 2011). These parallels reveal complex and divergent evolutionary lineages within the genus globally, while the new evidence clarifies the genetic makeup of a major *Casuarina* resource where the second largest plantation of this genus globally.

To elucidate the origin of *C. equisetifolia* in China and the parent source of the hybrids, we reconstructed genetic lineages along a hypothesized introduction pathway (Zhang et al. 2020; Luo et al. 2007). Although extensive gene flow and selection pressures may complicate ancestral signals from natural populations (Tricou et al. 2025; Jiang et al. 2024), phylogenetic analyses revealed that Chinese landraces form a monophyletic clade with Southeast Asian accessions, distinct from Australian natural provenances, likely supporting a historical expansion from Southeast Asia. This pattern maybe consistent with the shared subtropical climate and documented history of exchange between southern China and Southeast Asia. Previous field trials confirmed the fitness advantage of these introduced lineages, as Chinese and Southeast Asian germplasms exhibited higher survival and biomass accumulation than Australian provenances (Ye et al. 2004). Substantial gene flow between *C. equisetifolia* in China and Southeast Asia indicates that the parental source of the hybrids was primarily derived from this region. Based on these results, we propose encouraging both the preferential introduction of Southeast Asian germplasms and the use of artificial hybridization within China's germplasm enhancement projects.

There is no significant gene flow between *C. equisetifolia* in Southeast Asia and related species, indicating that hybrid lineages in China did not arise from the hypothetical external introductions. In contrast, extensive gene flow occurs between *C. equisetifolia* and *C. glauca* within China, suggesting that hybridization events have a distinct local origin and involve unique parental combinations. This maybe points to an adaptation‐driven mechanism: parental species first adapt independently to new environments before later contact leads to well‐adapted hybrid offspring. Geographically, *C. glauca* naturally inhabits higher latitude, cooler regions (Potgieter et al. 2014), while *C. equisetifolia* thrives in the warm, humid climates of Southeast Asia (Pinyopusarerk and Williams 2000). This niche divergence likely prevents *C. glauca* from establishing self‐sustaining populations in Southeast Asia. In China, however, *C. glauca* was introduced into regions with latitudinal and climatic conditions similar to its native range, bringing it into proximity with the *C. equisetifolia* landraces. This contact, combined with wind pollination and weak reproductive isolation in *Casuarina (*Varghese et al. 2004), fulfilled the necessary conditions for interspecific gene flow (Flores‐Renteria et al. 2025; Gamba and Muchhala 2023). And the frequent natural hybridization in introduced *Casuarina* zones supports this pattern (Gómez‐Rubio 2017).

### 4.2 Genetic Mechanisms of Adaptive Divergence and Breeding Implications

Shifts in allele frequency are usually associated with selection pressures and serve as a key indicator for understanding adaptive divergence in geographically separated populations (Buffalo and Coop 2020). Allele variations identified between CESC and CEN, as well as CGC and CGN, were primarily associated with plant immune responses to pathogens, suggesting that the initial introduction into the Western Pacific may have been accompanied by changes in disease resistance traits. According to plant–pathogen interaction theory, plants and microbiota usually establish an equilibrium in local habitats through coevolutionary processes (Ryalls and Harrington 2017). However, human‐mediated introductions for commercial or ecological purposes can disrupt this equilibrium, imposing new selective pressures (Young et al. 2017). Southeast Asia and southern China are among the regions with the highest precipitation globally, with warm, humid conditions promoting microbial activity, and potentially increasing the risk of plant disease outbreaks. For example, epidemiological reports indicate that *Casuarina* forests in Southeast Asia and China have been severely impacted by diseases caused by *Ralstonia solanacearum* (Ayin et al. 2019), whereas no comparable incidents have been reported in its native range to date. Furthermore, *Casuarina* species can symbiotically associate with specific ectomycorrhizal, endomycorrhizal fungi and Frankia that promote plant growth through nitrogen fixation and enhance disease resistance (Diagne et al. 2018). Changes in soil conditions after introduction may have altered interactions between *C. equisetifolia* and its symbiotic partners, driving genetic divergence (Gamba and Muchhala 2023). Beneficial plant‐microbiota symbioses are critical determinants of fitness in *Casuarina* species. This study elucidates the genetic variation mechanisms mediated by these symbioses, proposing a novel conceptual framework that extends beyond conventional cultivation methods and holds potential for application in future breeding programs.

Hybrid lineages can establish naturally only if they occupy unique ecological niches (Massatti et al. 2025). Their long‐term survival and evolutionary success depend critically on the ability to adapt genetically to new habitats and outperform their parental species in fitness (Zhao et al. 2015). In the present study, the absence of shifted SNPs shared between CESC vs. CEN or CGC vs. CGN suggests that genetic recombination, rather than subsequent allelic variation, was likely the primary driver of rapid adaptation. The hybrids were found to have inherited genes associated with RNA modification, protein phosphorylation, mRNA metabolism, and sucrose catabolism from *C. equisetifolia* , suggesting a potential hereditary basis for growth regulation and metabolic processes. In contrast, genes derived from *C. glauca* are primarily involved in cell wall remodeling and monosaccharide metabolism, which are critical processes for secondary metabolite synthesis that may play roles in pest/disease resistance. *C. equisetifolia* and *C. glauca* have successfully established in China, indicating their genetic capacity to adapt locally. Gene complementation between parental genotypes may enhance heterosis by activating additional genes, leading to better phenotypic performance (Hochholdinger and Yu 2025). For example, *C. glauca* usually exhibits superior resistance to diseases, pests, and waterlogging, while *C. equisetifolia* demonstrates significant advantages in growth rate, salt tolerance, and wind resistance (Zhang 2013). This genetic complementarity in Chinese hybrids indicates their capacity for sustained growth under regional stressors such as drought, poor soil quality, salinity, pests, diseases, and typhoons. This advantage is consistent with established adaptive traits observed in previous field experiment (Ye et al. 2004), providing valuable insights into the mechanisms underlying hybrid formation in China.

Early breeding programs in *Casuarina* have focused on species level testing, although some countries have used artificial crosses (Nicodemus et al. 2015). Pedigrees representing typical species characteristics remained a priority, whereas natural hybrid germplasms have often been overlooked due to incomplete background. This study confirms the existence of natural hybridization and domestication complexes, suggesting that the hybrids may have adaptive advantages in marginal habitats. Thus, rigid species concepts should be avoided in germplasm collection and selection, increasing recognition of individual accessions including the hybrids germplasms. Additionally, the present study indicates that the introduction of *C. equisetifolia* and *C. glauca* into the Western Pacific Rim may be associated with microbiota‐driven plant evolution, supporting the rationale for continued introductions from Southeast Asia to enhance genetic diversity in China. Furthermore, this study underscores that plant‐microbiota interactions, factors often overlooked in introduction and breeding programs, should be integrated into the strategic framework of *Casuarin*a breeding initiatives. Moreover, further research elucidating the mechanisms underlying root‐microbiota interactions is essential and holds significant potential for advancing selective breeding strategies.

Several limitations of this study merit careful consideration. The sampling strategy was primarily focused on three major *Casuarina* species widely cultivated in the Western Pacific Rim. Gene flow from other documented, albeit rare planted, congeneric species into the tested landraces cannot be excluded and warrants further investigation. A notable discrepancy emerged between analytical outcomes: phylogenetic analysis grouped *C. equisetifolia* and hybrids in China more closely with natural *C. glauca* than with the landrace, a finding that contrasts with results derived from FST and *D* statistic analyses. This inconsistency is likely attributable to the different analytical method (Kong and Kubatko 2021; Frankel and Ané 2023) or inherent limitations in the GBS data (Friel et al. 2021), underscoring the necessity for whole genome or pan‐genome sequencing approaches to resolve such phylogenetic conflicts. Furthermore, although a *Casuarina* genome has been published, the current reliance on *Arabidopsis*‐based functional annotations may obscure lineage specific metabolic pathways. This constraint highlights the importance of developing improved, genus specific genome annotations in future research.

## 5 Conclusions

Understanding the mechanisms driving the rapid adaptation of the *C. equisetifolia* in China is crucial for guiding future introduction and breeding efforts. This study compared the genetic structure of *C. equisetifolia* in China with reference provenances, revealing a potential phylogenetic history of gradual northward expansion of *C. equisetifolia* and hybridization with *C. glauca* exclusively in China. Shifts in SNP frequencies and associated genes suggest that pathogen resistance in *C. equisetifolia* and *C. glauca* has been varied since presence in the new habitat, and, the hybrid may have integrated several resistance related genes from *C. glauca* and photosynthesis related genes from *C. equisetifolia* , which elucidates the mechanisms of adaptive variation in these hybrids. These findings provide preliminary insights into the phylogenetic history, elucidate the genetic basis of local adaptation and underscore the value of natural hybrids, providing a foundational guide for future domestication and systematic breeding programs.

## Author Contributions

**Jingxiang Meng:** conceptualization (lead), data curation (lead), formal analysis (equal), funding acquisition (lead), investigation (lead), methodology (lead), project administration (equal), resources (equal), software (equal), validation (equal), visualization (equal), writing – original draft (lead), writing – review and editing (equal). **Yong Zhang:** project administration (equal), supervision (lead), writing – review and editing (equal). **Yongcheng Wei:** formal analysis (equal), resources (equal), software (equal). **Wei Lin:** data curation (supporting), formal analysis (equal).

## Funding

This research was funded by Funding by Science and Technology Projects in Guangzhou (2023A04J0710) and Fundamental Research Funds for the Central Non‐profit Research Institution of CAF (CAFYBB2020SY021).

## Conflicts of Interest

The authors declare no conflicts of interest.

## Supporting information


**Table S1:** Geographic information of all samples.

## Data Availability

I confirm that the Data Availability Statement is included in the main file of my submission, and that access to all necessary data files is provided to editors and reviewers. The raw data have been submitted to NCBI under submission number: PRJNA1287266.
