# Supplementary material for: Phylogenetic History and Adaptation Mechanisms of Casuarina equisetifolia Landraces in China
Source: Ecol Evol. 2026 Jan 5;16(1):e72781. doi: 10.1002/ece3.72781 (PMC12771583; doi:10.1002/ece3.72781)
Supplement: Supplementary file 1 — Table S1: Geographic information of all samples. [file ECE3-16-e72781-s001.docx]

Table S1 Geographic information of all samples

| Index | Species | Provenance | Individuals | Latitude | Longitude | Elevation |
| --- | --- | --- | --- | --- | --- | --- |
| 1 | *C. equisetifolia* | China (CEC) | sample2 | 18°40′N | 110°16′E | 2m |
| 2 | *C. equisetifolia* | China (CEC) | sample3 | 18°18′N | 109°25′E | 5m |
| 3 | *C. equisetifolia* | China (CEC) | sample4 | 18°21′N | 109°45′E | 2m |
| 4 | *C. equisetifolia* | China (CEC) | sample5 | 20°28′N | 110°32′E | 5m |
| 5 | *C. equisetifolia* | China (CEC) | sample6 | 18°40′N | 110°17′E | 5m |
| 6 | *C. equisetifolia* | China (CEC) | sample8 | 23°35′N | 117°8′E | 5m |
| 7 | *C. equisetifolia* | China (CEC) | sample9 | 18°30′N | 108°44′E | 7m |
| 8 | *C. equisetifolia* | China (CEC) | sample10 | 18°18′N | 109°25′E | 2m |
| 9 | *C. equisetifolia* | China (CEC) | sample12 | 20°28′N | 110°31′E | 3m |
| 10 | *C. equisetifolia* | China (CEC) | sample13 | 22°36′N | 113°51′E | 5m |
| 11 | *C. equisetifolia* | China (CEC) | sample14 | 22°48′N | 115°7′E | 3m |
| 12 | *C. equisetifolia* | China (CEC) | sample15 | 22°50′N | 115°15′E | 5m |
| 13 | *C. equisetifolia* | China (CEC) | sample16 | 22°49′N | 115°11′E | 5m |
| 14 | *C. equisetifolia* | China (CEC) | sample17 | 22°49′N | 115°8′E | 2m |
| 15 | *C. equisetifolia* | China (CEC) | sample18 | 22°52′N | 115°39′E | 5m |
| 16 | *C. equisetifolia* | China (CEC) | sample19 | 22°51′N | 115°44′E | 2m |
| 17 | *C. equisetifolia* | China (CEC) | sample20 | 22°51′N | 115°47′E | 3m |
| 18 | *C. equisetifolia* | China (CEC) | sample23 | 23°35′N | 117°8′E | 15m |
| 19 | *C. equisetifolia* | China (CEC) | sample24 | 25°27′N | 119°46′E | 5m |
| 20 | *C. equisetifolia* | China (CEC) | sample26 | 23°38′N | 117°25′E | 11m |
| 21 | *C. equisetifolia* | China (CEC) | sample28 | 18°40′N | 108°40′E | 5m |
| 22 | *C. equisetifolia* | China (CEC) | sample30 | 19°59′N | 110°47′E | 5m |
| 23 | *C. equisetifolia* | China (CEC) | sample32 | 24°55′N | 118°55′E | 5m |
| 24 | *C. equisetifolia* | China (CEC) | sample34 | 26°42′N | 120°7′E | 2m |
| 25 | *C. equisetifolia* | China (CEC) | sample35 | 26°42′N | 120°7′E | 5m |
| 26 | *C. equisetifolia* | China (CEC) | sample42 | 23°35′N | 117°8′E | 10m |
| 27 | *C. equisetifolia* | China (CEC) | sample43 | 20°15′N | 110°9′E | 5m |
| 28 | *C. equisetifolia* | China (CEC) | sample44 | 18°22′N | 109°45′E | 5m |
| 29 | *C. equisetifolia* | China (CEC) | sample45 | 19°8′N | 108°40′E | 6m |
| 30 | *C. equisetifolia* | China (CEC) | sample46 | 19°8′N | 108°40′E | 5m |
| 31 | *C. equisetifolia* | Malaysia(CESA) | sample29 | 5°55′N | 116°5′E | 0m |
| 32 | *C. equisetifolia* | Philippines(CESA) | sample11 | 12°21′N | 121°02′E | 121m |
| 33 | *C. equisetifolia* | Thailand(CESA) | sample21 | 12°33′N | 101°24′E | 101m |
| 34 | *C. equisetifolia* | Thailand(CESA) | sample22 | 9°21′N | 98°27′E | 98m |
| 35 | *C. equisetifolia* | Thailand(CESA) | sample27 | 7°10′N | 100°36′E | 100m |
| 36 | *C. equisetifolia* | Vietnamese(CESA) | sample39 | 18°24′N | 105°48′E | 105m |
| 37 | *C. equisetifolia* | Australia(CEN) | sample55 | 16°41′S | 145°34′E | 30m |
| 38 | *C. equisetifolia* | Australia(CEN) | sample60 | 12°39′S | 143°25′E | 1m |
| 39 | *C. equisetifolia* | Australia(CEN) | sample64 | 11°7′S | 132°20′E | 10m |
| 40 | *C. equisetifolia* | Australia(CEN) | sample65 | 11°7′S | 132°20′E | 10m |
| 41 | *C. equisetifolia* | Papua New Guinea(CEN) | sample66 | 9°17′S | 147°17′E | 10m |
| 42 | *C. equisetifolia* | Australia(CEN) | sample67 | 15°37′S | 136°19′E | 1m |
| 43 | *C. glauca* | Australia(CGN) | sample69 | 28°59′S | 153°23′E | 30m |
| 44 | *C. glauca* | Australia(CGN) | sample70 | 32°25′S | 152°19′E | 2m |
| 45 | *C. glauca* | Australia(CGN) | sample71 | 25°12′S | 152°37′E | 1m |
| 46 | *C. glauca* | Australia(CGN) | sample73 | 31°30′S | 152°40′E | 1m |
| 47 | *C. glauca* | Australia(CGN) | sample74 | 31°30′S | 152°40′E | 1m |
| 48 | *C. glauca* | Australia(CGN) | sample75 | 24°14′S | 151°52′E | 10m |
| 49 | *C. glauca* | Australia(CGN) | sample77 | 27°37′S | 152°51′E | 63m |
| 50 | *C. glauca* | Australia(CGN) | sample78 | 25°12′S | 152°37′E | 1m |
| 51 | *C. glauca* | China(CGC) | sample1 | 29°26′N | 121°57′E | 20m |
| 52 | *C. glauca* | China(CGC) | sample7 | 27°51′N | 121°9′E | 10m |
| 53 | *C. glauca* | China(CGC) | sample31 | 27°58′N | 120°48′E | 10m |
| 54 | *C. glauca* | China(CGC) | sample36 | 28°7′N | 121°1′E | 5m |
| 55 | *C. glauca* | China(CGC) | sample38 | 28°6′N | 121°1′E | 5m |
| 56 | *C. glauca* | China(CGC) | sample40 | 28°38′N | 121°31′E | 5m |
| 57 | *C. glauca* | China(CGC) | sample41 | 28°42′N | 121°23′E | 5m |
| 58 | *C. glauca* | China(CGC) | sample49 | 29°18′N | 121°52′E | 25m |
| 59 | *C. glauca* | China(CGC) | sample51 | 27°51′N | 120°50′E | 5m |
| 60 | *C. junghuhniana* | Indonesia(CJN) | sample25 | 8°55′S | 115°25′E | 1500 |
| 61 | *C. junghuhniana* | Indonesia(CJN) | sample33 | 8°13′S | 115°20′E | 1500 |
| 62 | *C. junghuhniana* | Indonesia(CJN) | sample48 | 9°51′S | 126°16′E | 800 |
| 63 | *C. junghuhniana* | Indonesia(CJN) | sample50 | 8°45′S | 115°15′E | 1500 |
| 64 | *C. junghuhniana* | Indonesia(CJN) | sample52 | 7°50′S | 111°47′E | 1500 |
| 65 | *C. junghuhniana* | Indonesia(CJN) | sample53 | 7°55′S | 112°55′E | 1600 |
| 66 | *C. junghuhniana* | Indonesia(CJN) | sample54 | 8°0′S | 113°35′E | 1500 |
